# Evaluation of the tuberculosis surveillance system in the Ashaiman municipality, in Ghana

**DOI:** 10.11604/pamj.2018.31.126.14993

**Published:** 2018-10-19

**Authors:** Rita Patricia Frimpong-Mansoh, Benedict Nii Laryea Calys-Tagoe, Esi Forewaa Therson-Coffie, Kwadwo Odei Antwi-Agyei

**Affiliations:** 1 Faculty of Public Health, Ghana College of Physicians and Surgeons, Accra, Ghana; 2 Ashaiman Municipal Health Directorate, Ghana Health Service, Ghana; 3 Department of Community Health, University of Ghana, School of Public Health-Legon, Ghana

**Keywords:** Evaluation, tuberculosis, surveillance, Ashaiman Municipality, Ghana

## Abstract

**Introduction:**

Tuberculosis (TB) was the leading cause of death from an infectious illness globally with an estimated 10.4 million new cases and 1.4 million deaths in 2015. In Ghana, from the 2013 TB prevalence survey conducted by the National Tuberculosis Control Programme, the incidence is estimated as 165 per 100,000 population and a mortality rate of 7.5 per 1,000 infected people. The Tuberculosis surveillance system is part of the general framework of the Integrated Disease Surveillance and Response. This evaluation was to assess whether the system is meeting its set objectives, assess its usefulness and describe its attributes.

**Methods:**

The TB surveillance system of the Ashaiman municipality was evaluated using Centre for Disease Control and Prevention updated guidelines for evaluating public health surveillance systems 2006. Records review from 2014 to 2016 was done to assess objectives of the system and surveillance data source of 2016 was used to assess attributes. Interviews were conducted at the various levels using semi-structured questionnaire and data analysis done with Epi info 7 and Microsoft Excel to run frequencies and percentages.

**Results:**

The surveillance system is well structured with standardized data collection tools. The system was found to be useful, though it just partially met its objectives. It was also found to be simple, flexible and fairly stable with average timeliness. It had low acceptability and is not geographically representative. It had low sensitivity of 45/100,000 and a low predictive value positive of 6.6%.

**Conclusion:**

The surveillance system was found to be useful but partially met its objectives. There is the need to improve the sensitivity, predictive value positive timeliness and acceptability.

## Introduction

Tuberculosis (TB) remains a major global health problem [1]. It causes ill-health in millions of people each year and is one of the top 10 causes of death worldwide [2]. Globally, an estimated 10.4 million people fell ill with the disease in 2015 with 1.4 million deaths [3]. The global pandemic of TB is growing as a result of the spread of Human Immuno Virus (HIV) infection, breakdown in health services and emergence of multidrug resistant TB [4, 5]. Many people who have been exposed to the bacilli and have strong immunity have latent TB infection and do not have symptoms [6, 7]. A number of factors make people more susceptible to TB infections. People who have HIV infection, chronic lung disease, smoking cigarettes, excessive drinking of alcohol and diabetics have an increased risk of developing tuberculosis [8-10]. The most important risk factor however is HIV [11]. Tuberculosis is closely linked to both overcrowding and malnutrition, making people in confinement e.g. prisoners [12] or those in hostels e.g. students, high risk groups. People who live in resource poor communities like slums and children who are in close contact with infected patients as well as health care workers attending to these patients are also at risk [13-15]. TB is more common among men than women and affects more people in the economically productive age group (15-59 years) [16]. The African Region has approximately one quarter of the world's cases, and the highest rates of cases and deaths relative to population [17]. Public health surveillance is a critical element in disease prevention and control, providing essential epidemiological data on which public health action can be based. Such data, not only identify areas of need for intervention, research and policy change, but can also be used to evaluate the impact of these actions [18]. Evaluation of public health programs is vital to ensure efficient program operation and, ultimately, health improvement. Surveillance evaluation seeks to ascertain whether a health event is monitored efficiently, and how well the purpose and objectives of the system is being met [19].

Tuberculosis prevalence survey conducted in Ghana by the National Tuberculosis Control Programme in 2013 revealed that there were 165 incident cases per 100,000 people. This is higher than World Health Organization (WHO) estimates of about 92 per 100,000 people [20]. The results showed that there were more undetected cases than previously estimated. The high prevalence of TB was as a result of the high number of TB cases among people living with HIV and Acquired Immune Deficiency Syndrome (AIDS). TB mortality rate in Ghana is considered high at 7.5 per 1,000 infected people [21]. To reduce this burden, detection and treatment gaps must be addressed, funding gaps closed and new tools developed. The socioeconomic burden of TB ranges from stigmatization from family and community to poverty. The Ashaiman municipality is one of the high incidence tuberculosis districts in the greater Accra region of Ghana based on a case notification rate of 72 per 100, 000 population in 2013 [22]. The objectives of the Ashaiman TB surveillance system is to early detect persons with infectious lung disease to improve chances of cure and reduce transmission of TB and to improve percentage of TB cases confirmed by microscopy. This evaluation was carried out from January to March 2017 to assess whether the system is meeting its set objectives, assess its usefulness and describe its attributes.

## Methods

This was a descriptive, evaluative study of the TB surveillance system using Centers for Disease Control and Prevention (CDC) updated guidelines for evaluating public health surveillance Systems (2006). The CDC updated guidelines is a standardized and validated guideline that is used to evaluate Public Health Surveillance Systems. The guideline provides the steps in evaluating the surveillance system. The CDC updated guideline 2006 was used because it was the most recent version available. The study was conducted in the Ashaiman municipal health directorate in the greater Accra region of Ghana from the 4^th^ January to 24^th^ March 2017. The population of Ashaiman for the year 2016 was 229,354 with a growth rate of 3.1%. The municipality is divided into seven (7) sub-municipal for the purposes of planning and delivery of health services; namely, Tsinaiagber, Mantseman, Maamomo, Amui Jor, Gbemi, Nii Man and Blakpatsona. Six out of the seven sub-municipalities have no functional public health facility. The municipality has one (1) Polyclinic, and seventeen (17) registered private clinics/hospitals and maternity homes and forty-one (41) Pharmacies and licensed chemical shops. There are six diagnostic TB centres and five treatment centres [23]. The study participants included 16 stakeholders who were identified by using purposive sampling technique based on their involvement in and relevance to the TB surveillance system. These stakeholders engaged in the evaluation of the surveillance system included the municipal director of health services, Medical Superintendent for Ashaiman polyclinic, district disease control officer, a medical officer at Ashaiman polyclinic, Direct Observed Therapy (DOT) corner nurses, district nutrition officer, institutional TB coordinators, biomedical scientists, laboratory technicians, community based surveillance volunteers, health information officer and the task shifting oficer. Face to face interviews were conducted to collect information regarding surveillance attributes by using a semi structured questionnaire based on CDC guidelines. The surveillance data source of the municipality for 2016 was used. Other relevant sources of information for this evaluation included a review of Ashaiman municipal health directorate annual reports on TB from 2014-2016 to assess targets of TB indicators. Sources of data reviewed were the district and facility TB registers, screening tool, monthly and quarterly reports, laboratory registers, treatment cards and District Health Information Management System (DHIMS II). Data obtained was entered into Microsoft Excel 2010 and analysed to generate frequencies and percentages. Incidence was calculated using National 2016 population distribution data for the Municipality. The attributes evaluated included: simplicity, timelines, acceptability, flexibility, representativeness, sensitivity, Predictive Value Positive (PVP), and usefulness. For analysis of sensitivity, positive predictive value, timeliness & representativeness, frequencies and percentages were used. Attributes with scores greater than 60% were ranked as good, those between 51% and 60% were ranked as average and those below 50% were ranked poor.

**Ethical issues:** This study was carried out as part of an operational research to determine the effectiveness of the TB surveillance system in the country and used mostly already existing health service records. Relevant administrative approvals were obtained from all the institutions involved before data was obtained.

## Results

**Purpose and operation of the system:** TB is a notifiable disease. The mandate of the National Tuberculosis Control Programme (NTP) is to provide leadership for the health sector response to fight tuberculosis in Ghana. It was launched in 1994 and aims at reducing the transmission of the disease to a level that is no longer a major public health problem. In terms of administration the Health Service is organized into a three-tiered system: National, Regional and District levels but is a five-tiered system in terms of service delivery: National, Regional, District, Sub-district and Community.

**Objectives of surveillance system:** The objectives for the TB surveillance system are: (1) to early detect persons with infectious lung disease to improve chances of cure and reduce transmission of TB; (2) To improve percentage of TB cases confirmed by microscopy. The indicators and targets of the surveillance system are: to detect at least 70% of TB cases in the District as smear positive; To attain cure rate of at least 85% of smear positive cases detected; To offer routine HIV testing to 100% of TB patients; To put at least 80% of HIV+TB patients on cotrimoxazole preventive therapy; To put at least 75% of TB+HIV on ART; To minimize adverse treatment outcome to below 2%.

**Case definition:** Case definition for suspected TB is any person with cough of 2 weeks or more. A confirmed case (smear positive) is a suspected person with at least 2 positive sputum smears for acid fast bacilli or one positive sputum smear with a chest x ray consistent with TB as determined by a treating medical officer. A confirmed smear negative TB is a suspected case who has 2 sets of sputum samples taken and negative for AFB on microscopy with chest x-ray findings consistent with TB. Or a patient who is severely ill with at least 2 sputum smears negative for PTB on microscopy with radiographic abnormalities consistent with extensive TB , a decision made by a clinician to treat with full course anti TB., or a patient who was initially smear negative but sample sent for culture tend out to be smear positive. However since the beginning of 2015, anyone who presents at the OPDs of the various facilities and especially those in the at risk population, are expected to be screened using a new TB questionnaire. Those who are strongly suspected are then sent to the lab (This became necessary because a lot of cases were missed using the WHO case definition above). All contacts of the confirmed cases (with or without cough) are also screened using the questionnaire.

**Minimum data and variables:** Patient information captured in the data include: The name, age, sex, date of registration, unit (facility) TB number, district TB number, residential address and telephone number of patient and of contacts, bacteriological results (sputum smear results as positive or negative, for pulmonary cases only), chest X-ray findings, patient classification (based on past history recorded as new case, transfer in, relapse, treatment failure, return after default and others like chronic or MDR TB), disease classification (anatomical site of disease-pulmonary or extra pulmonary) and treatment category. Every TB case is screened for HIV after counselling and the status recorded [including Co-trimoxazole Prophylaxis and Anti-Retroviral Therapy data]. Patient or case level data are available at the service delivery (DOTS centres) and district levels but only aggregate data are available at the Regional and National levels.

**Data collection and analysis:** The TB surveillance system is well structured with standardized data collection forms and tools. The district engages in both active and passive case search for tuberculosis cases as expected. Patients who present at the OPDs of the various facilities are screened using the new TB screening questionnaire. Suspected cases are sent to the laboratory with TB lab request form (TB05) and occasionally general laboratory request form for the sputum examination. Suspected cases produce 2 sputum samples at an hour interval and also do chest X-ray. However patients who are seen at facilities without the capacity to do TB investigations (sputum examination and/or x-ray) are referred to a diagnostic centre within the district for confirmation. Confirmed cases are then registered by the institutional TB coordinator or DOT corner nurse into the facility TB register (TB03) kept at the DOTS centres on tables and then fills the TB treatment cards (TB01). Each confirmed case is given a unique district number. Home verification is done before treatment is started. During such visits, contacts of smear positive TB patients (household members including children <5 years) are also screened. The TB patient is counselled with his/her selected treatment supporter (to help comply with treatment) and then put on treatment as soon as possible. The treatment support card is given to the treatment supporter for recording daily drug intake by the patient. Follow up sputum microscopy is done at months 2 and 5. The average time taken to confirm a suspected case of TB is one (1) day in the Municipality. A total of 103 cases were recorded in the district for 2016. This was made up of 3 children below 15 years and 100 adults. There were 73 males and 30 females.

**Figure 1:**
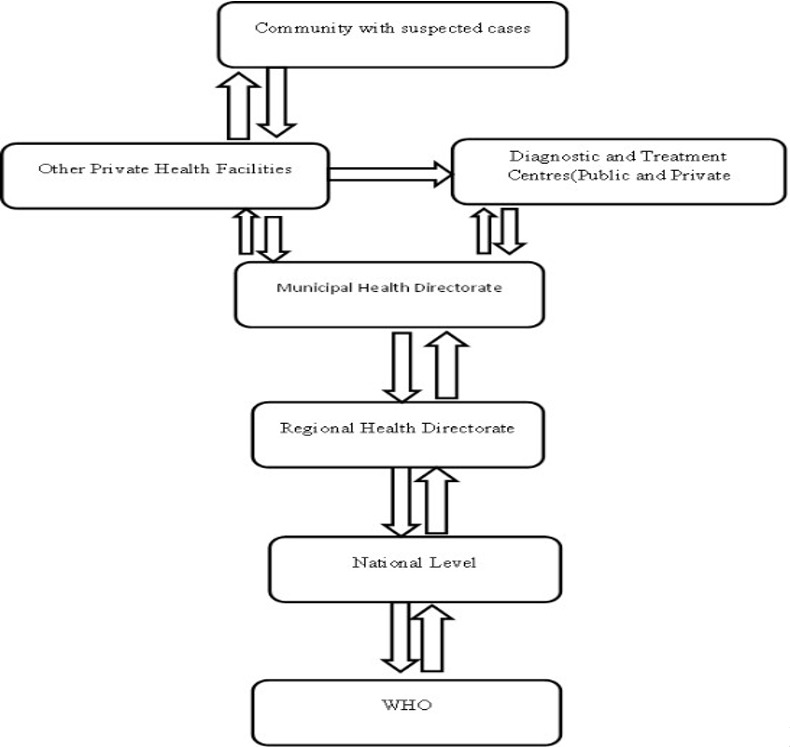
Data flow of Ashaiman TB surveillance system

**Data flow/dissemination** **Figure 1** **:** Data (hardcopy) are transmitted from the service delivery levels directly to the district level without going through the sub-districts (This is the norm in greater Accra and more especially Ashaiman where most of the diagnostic and treatment centres are in one sub-district). This is done monthly when the District TB coordinator visits the facilities to pick up the data. From the district level data are transmitted to the regional level quarterly (through email i.e. District Health Information Management System (DHIMS) and followed up with hardcopies) by the district TB coordinator. Data are sent from the regional level electronically to the national level quarterly by the regional TB M&E officers. The regional and national levels also receive data from some NGOs when necessary. The DHIMS forms for data entry was initially different from the NTP forms but that was corrected in 2015. The municipal TB coordinator receives data on the drugs given to patients from the polyclinic pharmacy only, since all the facilities collect their TB medication from there. The Regional External Quality Assurance Team (EQA) collects data from the TB04 (the laboratory TB registers) quarterly. The parameters used to assess sputum smear microscopy centres by EQA include sputum size (1-3mls), sputum thickness, cleanliness, sputum quality, microscopy results.

**Data validation and feedback:** Data validation is done at the district, regional and national levels. Feedback is given at periodic meetings, via social media (WhatsApp) or verbally.

**Resources used to operate the system:** There are both full time and part time personnel involved in the operations of the surveillance system. The full time workers include 2 DOT nurses, 1 task shifting officer, 1 laboratory personnel Part time personnel include 2 medical officers, 2 DOTS corner nurses, 1 biomedical scientist, disease control officer, community health nurse and community based surveillance volunteers. The materials used in the TB surveillance system are the tools for data collection (screening questionnaire, registers, laboratory request forms, monthly and quarterly report forms, laboratory reagents, slides, sputum containers etc.) and a DOT centre at the polyclinic. The polyclinic has a laptop, a microscope, and GeneXpert machine, provided by NTP. The principal funding agency for the system is the global fund through the NTP. The government of Ghana also contributes to the surveillance system by paying salaries of most of the workers. The private facilities directly pay their staff involved in the surveillance system.

### Performance of the system Table 1

**Usefulness of the surveillance system:** The TB surveillance system was found to be useful in detecting cases on time for accurate diagnosis, treatment and handling of contacts, providing estimates of the magnitude of morbidity and mortality related to TB and assessing the effect of the TB control program. The surveillance data has led to improved clinical practices (The direct involvement of 2 medical officers in the surveillance system to improve on case management).

**Table 1:** Assessment of some indicators of the TB surveillance system in Ashaiman Municipality

Indicators	Target (%)	Performance%		
		2014	2015	2016
Case detection (new smear positives)	70	74(110/154)	61(100/164)	65(67/103)
Cure rate (new smear positives)	85	88 (80/101)	89 (89/100)	-^Ý^
Default rate (all forms)	<1%	0	0	-^Ý^
Fatality rate (all forms)	<1%	2	0.6	-^Ý^
HIV screening	100	100	100	100

Ý Cure rate, defaulter rate and fatality rates for 2016 cohort is calculated ending 2017

### System attributes

**Simplicity:** Fifteen out of the 16 stakeholders interviewed described system as simple. (Case definition and TB algorithms were available at all DOT centres and easy to use, < 3minutes used to capture information on Screening tool).

**Data quality:** Assessed data quality was 73.7% (average of 14/19 cells completely filled in Facility TB registers). No discrepancies between facility and District registers was observed.

**Figure 2:**
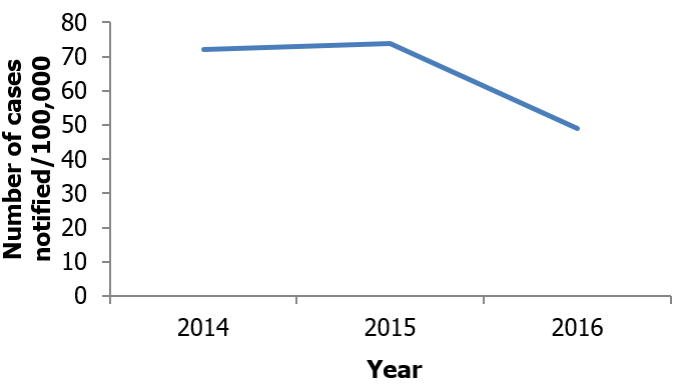
Trend of TB case notification in Ashaiman from 2014-2016

**Timeliness:** Forty out of 72(55.6%) reports were submitted to Regional level on time. Timeliness reflects the speed between steps in a public health surveillance system. The average time taken to confirm a suspected case of TB in the Municipality is one (1) day, but prior to suspicion of TB most patients are treated for other respiratory tract infections for quite some time. The District TB coordinator goes to all facilities and takes the monthly reports directly on the 5th of the ensuing month giving 100% timeliness of reporting from facilities .This is a strategy he came up with (from beginning of 2015) to solve the problem of late reporting from the private facilities earlier in the district. Quarterly reports are supposed to be submitted to the Region on or before 15th of the ensuing month in the next quarter but the District TB control officer is unable to send data on time. The District TB coordinator attributes this to the volume of work he has since his colleague Disease Control Officer left for School. The trend in TB notification between 2014 and 2016 is shown in Figure 2.

**Representativeness:** Four of the six treatment centers are located in one sub municipality.

**Acceptability:** Six out of 17 registered health facilities are TB diagnostic centers, 4/15 private health facilities have DOT centers. Other health workers are reluctant in accepting responsibilities in the Surveillance System.

**Sensitivity:** Following the national prevalence survey in 2013 the estimated incidence of TB is 165 per 100,000 population. Out of an expected 378 TB cases (using a population of 229, 354) the surveillance system detected 103 TB cases, a sensitivity of 27.2%, which is low. However the level of reporting from facilities to district was 100%.

**Flexibility:** The surveillance system was described by all stakeholders interviewed as flexible. There was a change of the screening tool from one screening form per person to one screening form for 30 people, the establishment of computer databases and electronic reporting; TB is now reported in the DHIMS. The surveillance system is integrated with the HIV surveillance system (Screening of all TB clients for HIV and vice versa). Nutritional assessment counseling and support (with various data collection forms) have also been incorporated into the TB surveillance system. Quarterly report forms on TB case registration (TB 07) and treatment results (TB 08) have been redesigned for easy data analysis. TB 07**A** and TB 08**A** are for adults whereas TB 07**P** and TB 08**P** are for children less than 15 years. There were regular meetings between Regional level and local-level officials (monitoring and evaluation and support visits) to discuss surveillance, conduct training, and discuss strategies to increase case detection and efficiency in the reporting system. These suggest that the system is flexible.

**Stability:** The TB surveillance system is able to collect, manage and provide data properly without failure and also the system is mostly operational when it is needed. Absence of enabler packages have however made home visits and home verification difficult.

Predictive Value Positive (PVP)

$$\text{PVP}=\frac{\text{Number}\;\text{of}\;\text{suspected}\;\text{cases}\;\text{confirmed}\;\text{at}\;\text{the}\;\text{lab}\;\left(\text{new}\;\text{smear}\;\text{positive}\;\text{cases}\right)}{\text{Total}\;\text{number}\;\text{of}\;\text{suspected}\;\text{cases}\;\text{referred}\;\text{to}\;\text{the}\;\text{lab}}$$

PVP=40 /609= 0.066. PVP=6.6% (expressed as percentage).

## Discussion

The findings of the evaluation shows that the TB surveillance system of Ashaiman municipality is well structured with specific roles assigned to different stakeholders and with good channels of communication. This was demonstrated in the flow of information and the levels of reporting as well as the feedback from the district and regional levels. The results are comparable to other TB surveillance systems in the country [23, 24]. A public health surveillance system is useful if it contributes to the prevention and control of adverse health-related events and also contributes to performance measures. The TB surveillance system was found to be useful as it resulted in the assignment of two medical doctors to the DOT centre to help improve case management, incorporation of TB screening in community screenings organized by health staff and inclusion of health Education on TB at all fora organized by health staff . The improvement in defaulter rate could be partly explained by these. Overall good data quality observed showed the level of commitment of the personnel directly involved in the surveillance system. Most patients interviewed were satisfied with the good attitude of focal persons towards them. HIV screening was done for all TB clients indicating how TB is well integrated with the HIV surveillance. Integrating TB and HIV promoted good treatment outcomes and this was clear in the zero death rate recorded in 2016. TB/HIV co-infections are now being managed well at the facilities as a result of integration of the TB surveillance system with that of HIV. This is similar to the situation in the entire country as presented by the National TB Control Programme. The proportion of TB clients counselled and tested for HIV has risen steadily from 17% in 2006 to 82.7% in 2015 [25]. The surveillance system also had some challenges. Acceptability is generally low. Of the fifteen private facilities, only four have DOT centers and some of the private facilities had threatened to stop providing surveillance data. This may be due to the fact that private health facilities are profit oriented but TB diagnosis and treatment is free. This means that the staff that are paid at the private facilities would use the man hours to provide free service for TB at the expense of income generating services. Again all the staff at private facilities are paid directly by their employers. Low acceptability of TB surveillance system from Private Health facilities is common in other parts of the country [25].

Representativeness is also low. Four(4) out of the six(6) treatment centres in the municipality are located in one sub-district and this means that clients have to travel some distance before assessing TB services. The sensitivity of the surveillance system is low. This is similar to the situation in other districts in the Greater Accra Region and the country as a whole [24, 25]. The National prevalence survey in 2013 estimated TB incidence in Ghana to be 165/100,000 population. This means that with a population of 229, 934, case detection for 2016 in the Municipality should have been 378 but only 103 cases were detected. Several undetected cases mean that the aim of the Tuberculosis Control Programme to end the pandemic by 2030 would be far from being attained. The surveillance system, at the district level, directly involves health facilities instead of Sub municipalities and this partly accounts for work load on district TB coordinator leading to his late reporting in DHIMS. The ability of a surveillance system to collect, manage and provide data properly without failure makes it stable. The stability of the surveillance system is threatened by the lack of enabler packages for clients (adults who do not have MDR) as well as staff. This, staff claim, makes case management difficult because more often staff have to support clients from their personal funds and also due to lack of funds home visits and contact tracing have become quite difficult. Predictive Value Positive was 6.6 %. This may partly confirm the era of smear negative TB cases which are detected by the GeneXpert machine. With the introduction of the GeneXpert machine it is expected that case detection would be increased.

## Conclusion

The tuberculosis surveillance system in the Ashaiman Municipality is well structured with standardized data collection tools. The system was found to be partially effective in meeting its objectives. It is useful as it has led to improved clinical practices, flexible, simple and of good data quality but needs to improve on sensitivity, acceptability, timeliness and predictive value positive. It was found to be useful and has led to improved clinical practices and informed decision making. It is recommended that to improve on case detection more screening activities should be carried out in communities, within facilities and departments. The district health management Team should encourage private facilities to be involved in TB surveillance as well as get a deputy for the district disease control officer to support surveillance activities. In the absence of enabler's package, facilities should support sputum transport, home verification and home visits. This can be done when surveillance activities are budgeted for.

### What is known about this topic

- It is well known that the tuberculosis surveillance system is necessary for the early detection of cases, improvement in the cure rate and reduction in the transmission of TB.

### What this study adds

- The TB surveillance system in Ashaiman is well structured and robust. The flow of information from one level of the system to the other is good enhancing its flexibility. The surveillance system is integrated with the HIV surveillance system. This was seen in the screening of all TB clients for HIV;
- Some of the main gaps identified are the low representativeness and acceptability from Private health care providers as well as low sensitivity and Predictive value positive.

## Competing interests

The authors declare competing interests.
